# Distinct pattern and prevalence of *Plasmodium falciparum* dihydropteroate synthase gene mutations in children with sickle cell anaemia and haemoglobin AA in Benin City, Nigeria: the impact of HbAA

**DOI:** 10.11604/pamj.2022.43.80.34334

**Published:** 2022-10-13

**Authors:** Izehiuwa Gertrude Enato, Ayebo Evawere Sadoh, Okoeguale Michael Ibadin, Magdalene Erhieyouvbe Odunvbun, Iriagbonse Iyabo Osaigbovo

**Affiliations:** 1 Edo State University, Uzairue, Edo State, Nigeria,; 2 Institute of Child Health, University of Benin, Benin City, Edo State, Nigeria,; 3 Department of Child Health, University of Benin Teaching Hospital, Benin City, Edo State, Nigeria,; 4 Department of Medical Microbiology, University of Benin, Edo State, Nigeria,; 5 Department of Medical Microbiology, University of Benin Teaching Hospital, Edo State, Nigeria

**Keywords:** Molecular markers, sulphadoxine resistance, *Plasmodium falciparum* dihydropteroate synthase

## Abstract

**Introduction:**

specific mutations on the Plasmodium falciparum dihydropteroate synthase (Pfdhps) gene mediate sulphadoxine/pyrimethamine (SP) resistance and thus, pose a threat to the efficacy of SP-Intermittent Preventive Therapy (SP-IPT) in malaria chemoprevention in children, including those with sickle cell anaemia (SCA). This study determined the distinct pattern and prevalence of Pfdhps mutations in children with SCA and in those with homozygous haemoglobin A (HbAA) in Benin City, Nigeria; showing the impact of haemoglobin phenotype.

**Methods:**

this was a cross-sectional study involving children with SCA and HbAA. Those with successfully amplified Pfdhps genes were included in the study. Point mutations and mutant haplotypes of the Pfdhps gene were identified. Parasite density (PD) was determined by estimating the parasite numbers/μl of blood from the thick film. Descriptive, univariable and multivariable analysis were used appropriately.

**Results:**

a total of 146 children: 71 with SCA and 75 with HbAA were recruited, with a mean age of 46.6 ± 13.0 and 36.4 ± 17.6 respectively; proportion of males were 45(63.4%) and 43(57.3%) respectively. I431V, S436A, A437G, A581G, and A613G mutations were present; but the K540E mutation was absent. ISGKAA 41(28.1%) and VAGKGS 61(41.8%) were the most prevalent mutant haplotypes in this study. The prevalence of VAGKGS haplotype 43(57.3%) was significantly higher in HbAA group compared to that 18(25.4%) in the SCA group (p < 0.001). The prevalence of ISGKAA in SCA group 25(35.2%) was significantly higher than that 16(21.3%) in the HbAA group (p=0.032). HbAA phenotype was the only significant predictor for the presence of the VAGKGS mutant haplotype (aOR: 3.0, 95%CI: 1.375 to 6.499; p=0.006).

**Conclusion:**

the HbAA phenotype was a significant predictor for the occurrence of the quintuple mutant haplotype (VAGKGS). The K540E mutation was absent; thus, SP-IPT can be explored in children younger than five years with SCA.

## Introduction

In children with sickle cell anaemia (SCA), Sulphadoxine/Pyrimethamine as Intermittent Preventive Treatment (SP-IPT), has been shown to be efficacious and effective in reducing malaria induced morbidity and mortality [1,2]. However, point mutations on *Plasmodium falciparum dihydrofolate reductase (pfdhfr)* and *Plasmodium falciparum dihydropteroate synthase (pfdhps)* genes pose a threat to the efficacy of SP-IPT; these mutations form the molecular markers for sulphadoxine/pyrimethamine (SP) resistance [3]. Molecular markers for pyrimethamine resistance were established long before SP resistance [4]; the *pfdhfr* mutations have become fixed in the general population [5,6]. SP resistance was heralded by point mutations on the *P. falciparum dihydropteroate synthase (pfdhps)* gene at codons 436 (S436A), 437 (A437G), 540 (K540E), 581 (A581G), and 613 (A613S/T) [7-10].

SP-IPT (in infants and young children) is said to remain efficacious in areas where the prevalence of *pfdhps* K540E is less than 50%, even in the presence of significant SP resistance and malaria treatment failure, which is indicated by the quintuple mutant haplotype: 3DHFR (N51I+C59R+S108N) + 2DHPS (A437G+K540E) [11-13]. A high prevalence (50% or more) of *pfdhps* K540E mutant allele in a region, is indicative of failure of SP as IPT in those with little or no malaria immunity [11-13]. Thus, SP-IPT can be recommended in regions with low prevalence (<50%) of *pfdhps* K540E mutations [11]. A few studies have described an emerging *pfdhps* I431V mutation and *pfdhps* quintuple mutant haplotype VAGKGS (I431V, S436A, A437G, K540 A581G and A613S) [6,14,15].

White *et al*. and Pongtavornpinyo *et al*. mathematical models, further described that mutations such as *pfdhps* mutations on malaria parasites are statistically more likely to occur and spread from infections with a large parasite biomass [16,17]. Infants and young children with normal haemoglobin phenotype (HbAA) have a higher parasite biomass/density compared to their counterparts with homozygous haemoglobin S (HbSS) as seen in those with SCA, with a lower parasite biomass [18,19]. Therefore, the occurrence and spread of mutant genes (thus SP resistance) may be higher in infants and young children with HbAA compared to those with sickle cell anaemia. The aim of this study was to determine and compare the prevalence and distinct pattern of *pfdhps* mutations in children aged 6-59 months with SCA and those with HbAA in Benin City, Nigeria; showing the impact of haemoglobin phenotype (HbAA and HbSS: SCA) and parasite density.

## Methods

### Study design and setting

This was a cross-sectional comparative study, which was carried out at the University of Benin Teaching Hospital (UBTH), Sickle Cell Centre (SCC) and Children´ Clinic at The Central Hospital all in Benin City, Edo State, Nigeria. Edo State is located in the South-south geographical region of Nigeria, where malaria transmission is holoendemic and stable. UBTH, Benin City provides primary, secondary and tertiary health care services to the people living in the immediate five Local Government Areas of Egor, Ikpoba-Okha, Oredo, Ovia North-East and Ovia South-West of Edo State. Sickle Cell Centre (SCC), is attached to the Central Hospital, Benin City. SCC provides secondary health care services to population within and outside Benin City. The SCC manages only sickle cell disease (SCD) patients (children and adults); clinics are run daily.

### Sample population

Children with SCA aged 6-59 months were recruited from the sickle cell clinic in UBTH and the Sickle Cell Centre attached to the Central Hospital, Benin City; while children with HbAA aged 6-59 months were recruited from The General Practice Clinic of UBTH, and Children´ clinic of the Central Hospital, Benin. Minimum sample size was calculated using the formula for comparing two proportions (HbAA and SCA groups) [20], using 95% confidence interval (CI). The minimum sample size was 164 for each study group.

**Inclusion criteria:** children aged 6-59 months with SCA with Haemoglobin SS phenotype or haemoglobin AA phenotype (confirmed by electrophoresis using cellulose acetate paper); and with *P. falciparum* parasite seen on blood smear determined using microscopy.

**Exclusion criteria:** all children on sulpha-based medication/chemoprophylaxis, such as trimethoprim/sulphametoxazole. This information was obtained from the care givers/parents. Study participants were recruited following screening of all children aged 6-59 months visiting the respective clinics. Patients who met the inclusion criteria were recruited consecutively until sample size of 164 for each study group was met.

**Data collection:** at screening and recruitment, a proforma was used to obtain information from the parents or guardians on the child´s age, date of birth, and sex; information on drug history, including use of sulpha-based medication such as co-trimoxazole -trimethoprim/sulphamethoxazole. Three millilitres of blood was aseptically obtained from peripheral veins of the participants for Haemoglobin electrophoresis (to determine/confirm the Haemoglobin genotype of study participants); thick and thin blood film for malaria parasite and parasite density. Blood was collected on filter paper for dried blood spot (DBS). At the end of sample collection, the filter paper dried blood spots positive for P. falciparum following screening by microscopy, were selected and transported in a desiccant via courier to the London School of Tropical Medicine and Hygiene (LSTMH), United Kingdom. Parents/guardians were counseled following results of microscopy and haemoglobin genotype for malaria parasite. All patients with malaria parasitaemia received prescription for Artemisinin based Combination Therapy (ACT), irrespective of the absence/presence of symptoms.

### Laboratory analysis

#### Determination of Haemaglobin phenotype

This was done using cellulose acetate method at pH 8.4. This took place in the Medical Haematology Laboratory, University of Benin Teaching Hospital and the Haematology Laboratory of the Central Hospital, Benin. One milliliter of blood collected from each child was transferred into a test tube. The red blood cells were washed in 0.9% normal saline by adding 9mls of normal saline to 1 ml of blood in the test tube. The solution was then centrifuged at 5000rpm for 5 minutes. The supernatant was discarded, leaving the cells. Three milliliters of water was then added to 1ml of the cells to cause lysis of the red cells. With the use of a pipette (GIBSON, made in France) and a disposable plastic pipette, 0.1ml of the resultant solution (haemolysate) was applied to the cellulose acetate paper and the paper then placed in the electrophoresis chamber for 30 minutes. The rate of migration on the electrophoretic machine (Beckman electrophoresis machine Model R-120, made in Germany) was used to determine the haemoglobin genotype.

*Thick and thin blood film for malaria parasite and speciation using Geimsa stain:* i) Each clean grease-free slide was labelled with the date and the sample number, age and sex of the patient. ii) Using a pipette (GIBSON, made in France) and a disposable plastic pipette tip, a drop of blood from the subject was made at one end of the labelled slide. iii) A smear of blood was then made (using the plastic cap of the needle) with the drop of blood, covering an area of 15X15 mm. iv) On the same slide, a drop of blood was applied 10 mm away from the thick smear. v) The blood smear was spread using the smooth edge of a slide spreader to make a thin film of blood. To make the thin film of blood, the slide spreader was placed at 45 degrees to the horizontal just behind the blood spot. This was to allow the blood spread by capillary action against the edge of the slide spreader. Then with a single smooth swipe, the spreader was moved towards the edge opposite the thick film so that a thin film with a “tail” is formed. vi) The slide containing the thick and thin blood was allowed to air-dry. vii) After thorough drying, the slide was placed horizontally on a staining rack. Viii) A small drop of methanol was then applied to the thin film. The thin film was then allowed to fix for one minute. ix) After fixing the thin blood smear, the slides were placed facing downwards in a staining trough. x) The Geimsa stain was then slowly poured into the staining trough and allowed to stand for 30 minutes using 3% Geimsa solution (freshly prepared). xi) After 30 minutes, the stain was flushed off the slides with clean tap water. xii) The slide was then allowed to air dry. xiii) When the thick film was completely dry, a drop of immersion oil was then applied on the film. xiv) The oil was spread to cover an area about 10 millimeters in diameter. xv) Using 100X objective magnification, the smear was examined for malaria parasites and pigments. The result was then reported as positive if malaria parasite was seen. If no malaria parasite seen, it was reported as NPF (No parasites found). xvi) The different plasmodium species were identified on the thin film using 100X objective to examine for the parasites.

#### DNA Extraction, nested PCR amplification and direct sequencing

The analysis was carried out in stages; DNA extraction from bloodspots on ?lter paper was carried out first using the Chelex method in a 96-well plate format as described by Plowe *et al*. in 1995 [21]. DNA extraction was followed by nested PCR amplification of the *pfdhps* genes. 711 bp products of *pfdhps* genes were sized against 100 bp molecular weight marker on 1.2% agarose gel stained with ethidium bromide. Exonuclease I-Fast Alkaline Phosphatase was used to enzymatically purify PCR products, according to the manufacturer´s instructions, followed by direct sequencing of products. Point mutations at codons 431, 436, 437, 540, 581 and 613 of the *pfdhps* gene were read and recorded using Chromas software 2.4.

*Determination of parasite density (degree of parasitaemia):* counting parasite numbers was done by estimating parasite numbers/µl of blood from the thick film. i) The average number of parasites per HPF was counted on the thick film. Twenty fields were examined to determine the average number of parasites per HPF. ii) The number of parasites counted per HPF was multiplied by a factor of 500. The computed value was parasite numbers/µl of blood (parasitaemia) for the individual. Parasite density =250 000 parasites/µl of blood is one of the laboratory criteria for severe malaria.

### Definition of variables

*Dependent variables: Pfdhps* point mutations: *Plasmodium falciparum* dihydropteroate synthase point mutations at codons 431, 436, 437, 540, 581 and 613 (I431V, S436A, A437G, K540E, A581G and A613S respectively). *Pfdhps* mutant haplotypes: *Plasmodium falciparum* dihydropteroate synthase haplotypes; a combination of point mutations (single, double, triple, quadruple and quintuple mutant haplotypes).

*Independent variables:* SCA/HbSS: sickle cell anaemia/ Homozygous Haemoglobin SS; HbAA: Homozygous Haemoglobin AA; GMPD: Geometric Mean Parasite Density - parasite density; Age: 6-59 months; Gender: Male and Female.

### Statistical analysis

All data generated were collated, checked and analysed using statistical package for social sciences (SPSS) version 17.0 (SPSS for Window Inc; Chicago, LL, USA). Gender distribution and prevalence of *Pfdhps* point mutations and mutant haplotypes were recorded as simple percentages. Continuous variables (age and parasite density) were recorded as means and standard deviation. Logarithmic transformation was applied to all values recorded for parasite density to make the data normally distributed and amenable to statistical analysis. Parasite density was recorded as geometric mean parasite density (GMPD). Test of association between variables, such as prevalence of *Pfdhps* point mutations/mutant haplotypes in children with SCA and HbAA was done using Chi-square. The GMPD between two groups: (those with the presence and those with absence of *Pfdhps* point mutations/mutant haplotypes) was compared using independent T-test. Mean difference across more than two categories of variables (GMPD across mutant *Pfdhps* haplotypes) was done using one-way Analysis of Variance (ANOVA). Multiple logistic regressions model was used to identify predictors (Haemogblobin phenotype and Parasite density) of molecular markers of resistance. Statistical significance was set at p < 0.05.

**Ethical consideration:** ethical approval for the study was obtained from the Ethics and Research Committee of University of Benin Teaching Hospital (UBTH), Benin City; UBTH (ADM/E22/A/VOL.VII/839). A written informed consent was obtained from parents and/or guardians of the study participants by the investigators before samples were collected.

## Results

A total of 164 children with SCA and 164 children with HbAA, who had *Plasmodium falciparum* parasites/isolates demonstrated using microscopy, were recruited for this study. However, following DNA Analysis of dried blood spots, only 71 (43.3%) children with SCA and 75 (45.7%) children with HbAA had successfully amplified *P. falciparum dhps* genes.

### Age and sex distribution of study participants

Of the 71 children with SCA and 75 children with HbAA and amplified *P.falciparum dhps* gene, males were 45(63.4%) and 43(57.3%) respectively (Table 1). The mean age ±SD of children with amplified *Pfdhps* in the SCA group 46.6±13.0 was statistically significantly higher than that 36.4±17.6 observed in the HbAA group (t=3.92, p < 0.001). The characteristics of both study groups are shown in Table 1.

**Table 1:** age and gender distribution of study population

Characteristics	SCA (n=71) n(%)	HbAA (n=75) n(%)	χ2	p
**Gender**				
Male	45(63.4)	43(57.3)	0.455	0.557
Female	26(36.6)	32(42.7)		
**Age Group (in months)**				
6-11	3(4.2)	11(14.7)	13.498	0.009
12-23	3(4.2)	10(13.3)		
24-35	7(9.9)	11(14.7)		
36-47	6(8.5)	9(10.0)		
48-59	52(73.2)	34(45.3)		

### Pattern and prevalence of mutant *pfdhps* point mutations and *pfdhps* haplotypes

The *pfdhps* point mutations found, and their prevalence were I431V 82/146 (56.2%), S436A 94/146 (64.4%), A437G 144/146 (98.6%), A581G 83/146 (56.8%) and A613S 83/146 (56.8%) (Table 2). The K540E mutation was totally absent in this study. The different mutant *pfdhps* haplotypes identified in the study were single mutant haplotype: ISGKAA 41(28.1%) and IAAKAA 2(1.4%); the double mutant haplotype: IAGKAA 5(3.4%) and ISGKGA 4(2.75); triple mutant haplotype: IAGKAS 7(4.8%), IAGKGA 1(0.7%), ISGKGS 1(0.7), VAGKAA 1(0.7%), and VSGKGA 1(0.7%); quadruple mutant haplotype: IAGKGS 2(1.4%), VAGKGA 5(3.4%), VAGKAS 3(2.1%); and the quintuple mutant haplotype: VAGKGS 61(41.8%), (Table 2). Each of these *pfdhps* mutant haplotypes was found in both groups (SCA and HbAA), except the IAAKAA, VAGKAS, VSGKGA, VAGKAA haplotypes which were found only in SCA group and the ISGKGA found only in HbAA group (Annex 1).

**Table 2:** prevalence of *Pfdhps* point mutations/mutant haplotypes and their association with mean parasite density

*Pfdhps* point mutation/ Haplotype	PRESENT GMPD±SD* n(%) (parasite/µl)	ABSENT GMPD±SD* n(%) (parasite/µl)	t	p-value	95% CI
**Point mutations**					
I431V	82 (56.2) 1842.47±12.32	64 (43.8) 1022.12±6.59	-1.6	0.120	0.26 to 1.17
S436A	94 (64.4) 1857.80±8.26	52 (35.6) 878.82±8.26	-1.9	0.056	0.22 to 1.02
A437G	144 (98.6) 1388.67±9.73	2 (1.4%) 8943.35±1.17	1.2	0.250	0.27 to 156.81
A581G	83 (56.8) 2184.24±12.57	63 (43.2) 809.28±5.67	-2.7	0.008	0.18 to 0.77
A613S	83 (56.8) 2143.88±11.19	63 (56.8) 829.28±1.28	-2.5	0.012	0.19 to 0.81
**Mutant haplotypes**					
ISGKAAa	41(28.1) 694.54±10.99	105(71.9) 1942.67±10.99	2.4	0.016	1.21 to 6.46
VAGKGSb	61(41.8) 2764.39±13.12	85(58.2) 846.45±6.46	-3.1	0.003	0.82 to 0.65
Double c	9(6.2) 2157.25±9.21	137(93.8) 1368.04±10.00	-0.6	0.567	0.13 to 3.05
Tripled	11(7.5) 554.37±10.28	135(92.5) 1536.38±10.29	1.4	0.159	0.67 to 11.49
Quadruplee	10(6.8) 815.46±4.80	136(93.2) 1476.05±10.38	0.8	0.433	0.41 to 8.06

*All mixed samples n=12(8.2%) and samples with wild type variant *pfdhps* haplotype n=2(1.4%): IAAKAA were excluded from the analysis of association between GMPD and Prevalence of *Pfdhps* point mutations/haplotypes. GMPD:Geometric mean parasite density aSingle mutant haplotype: ISGKAA, IAAKAA bQuintuple mutant haplotype: VAGKGS cDouble mutant haplotype: IAGKAA, ISGKGA dTriple mutant haplotype: IAAKGS, IAGKAS, IAGKGA, ISGKGS, VAGKAA, VSGKGA eQuadruple mutant haplotype: IAGKGS, IAGKGS, VAGKGA, VAGKAS IAAKAA: Wild type variant MIXED: samples containing parasite strains with multiple mutant *pfdhps* haplotypes

### Pattern and prevalence of *pfdhps* point mutations and mutant haplotypes in children with SCA and in those with HbAA

**Figure 1:**
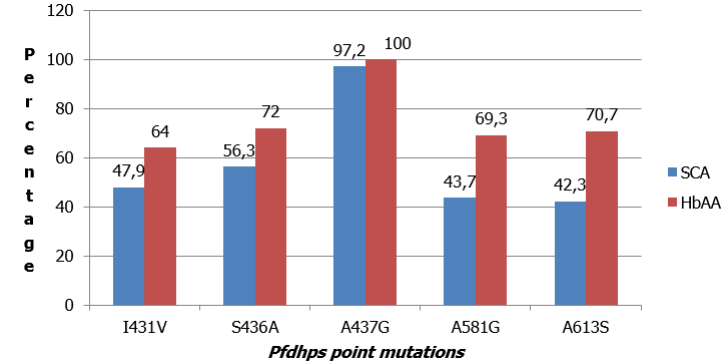
prevalence of *pfdhps* point mutations in SCA and HbAA groups

**Figure 2:**
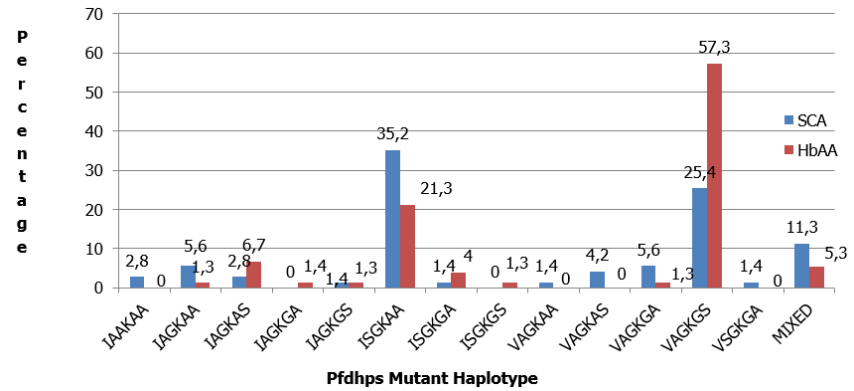
prevalence of *pfdhps* haplotypes in SCA and HbAA groups

The prevalence of S436A: 54(72.0%), A581G: 52(69.3%) and A613S: 53(70.7%) mutations in HbAA group were statistically significantly higher than 40(56.3%), 31(43.7%) and 30(42.3%) respectively found in the SCA group (p=0.048 for S436A, p=0.002 for A581G; and p=0.001 for A613S) (Figure 1). The point mutation with the highest prevalence in the study population was the A437G mutation; with a prevalence of 69(97.2%) in SCA group, which was lower than 75(100%) in the HbAA group (p=0.235). Also, the prevalence of I431V mutation 48(64.0%) was higher in HbAA group than in SCA group 34(47.9%) (p=0.050). The predominant *pfdhps* haplotypes of parasites infecting children in both study groups were the single mutant haplotype: ISGKAA and the quintuple mutant haplotype: VAGKGS. In the SCA group, the prevalence of ISGKAA *pfdhps* mutant haplotype: 25(35.2%) was statistically significantly higher than 16(21.3%) found in HbAA group (p=0.032). In the HbAA group, the prevalence of VAGKGS *pfdhps* mutant haplotype 43(57.3%) was statistically significantly higher than 18(25.4%) found in SCA group (p < 0.001). There was no statistical significant difference in the prevalence of the other mutant haplotypes between both groups (Figure 2).

### Prevalence of *pfdhps* mutant haplotype and *pfdhps* point mutations and their association with the degree of parasitaemia (GMPD)

The Geometric Mean Parasite Density (GMPD) was higher in all samples with *pfdhps* point mutations compared to those without each of the mutations; but the GMPD was only significantly higher in children with *pfdhps* A581G (2184.24 ± 12.57) and A613S (2143.88 ± 11.19) mutations than in those without A581G and A613S mutations (p=0.008 and p=0.012) respectively. The GMPD (2764.39±13.12) was only significantly higher in samples with VAGKGS mutations compared to that (846.45±6.46) in those without VAGKGS haplotype (p=0.003) (Table 2). On the contrary, the GMPD was significantly higher in samples without the ISGKAA mutant haplotype (1942.67±10.99) compared to that (694.54±10.99) in those with ISGKAA haplotype (p=0.016). The GMPD differed significantly across samples with the different mutant haplotypes (p=0.016); being highest in those with the quintuple mutant haplotype VAGKGS (2764.39±13.12) (Annex 2). The post hoc test showed that the GMPD in those with *pfdhps* quintuple mutant (VAGKGS) haplotype was significantly higher than (694.54 ± 10.99) in those with *pfdhps* single mutant haplotypes (ISGKAA).

### Multiple binary logistic regressions model for the effect of degree of parasitaemia (geometric mean parasite density) and haemoglobin phenotype on the prevalence of *pfdhps* point mutations/haplotypes

Multiple logistic regressions model was constructed for the effect of parasite density and haemoglobin phenotype on the presence of *pfdhps* point mutations and *pfdhps* mutant haplotypes. HbAA phenotype was the only significant predictor for the presence of A581G (aOR 2.3, 95% CI: 1.105 to 4.851; p=0.026) and A613S point mutations (aOR 2.7, 95% CI: 1.293 to 5.745; p= 0.008). Parasite density was not a significant predictor for A581G and A613S point mutations (Table 3). Similarly, a significant relationship was found between HbAA phenotype and VAGKGS haplotype, which is three (3) times more likely to occur in persons with HbAA phenotype (aOR 3.0, 95% CI: 1.375 to 6.499; p=0.006) (Table 3).

**Table 3:** multiple binary logistic regressions model for the effect of degree of parasitaemia (geometric mean parasite density) and haemoglobin phenotype on the prevalence of *pfdhps* point mutations/haplotypes

*Pfdhps* point mutation/ Haplotype	β	p-value	Adjusted Odds Ratio (aOR)	95% CI
**Point mutations**				
**A581G**				
HbAA	0.84	0.026	2.3	1.105 - 4.851
Parasite Density	0.30	0.140	1.4	0.907 - 1.996
**A613S**				
HbAA	1.003	0.008	2.7	1.293 - 5.745
Parasite Density	0.240	0.233	1.3	0.856 - 1.889
**Mutant haplotypes**				
**ISGKAA**				
**HbAA**	0.590	0.158	1.8	0.795 - 4.089
**Parasite Density**	0.375	0.100	0.687	0.439 - 1.075
**VAGKGS**				
HbAA	1.095	0.006	3.0	1.375 - 6.499
Parasite Density	0.348	0.09	1.4	0.947 - 2.118

## Discussion

An important finding in this study is the distinct pattern and prevalence of the *pfdhps* point mutations: I431V, S436A, A437G, A581G, and A613S and mutant haplotypes identified in this study; the prevalence of each point mutation was higher in children with HbAA compared to those with SCA. The predominant *pfdhps* mutant haplotypes found in this study were VAGKGS (which was significantly higher in children with HbAA) and ISGKAA (which was significantly higher in children with SCA). Haemoglobin phenotype AA was a significant predictor for the occurrence of VAGKGS. The presence of the emerging *pfdhps* I431V, and the high prevalence of *pfdhps* A581G and A613S mutations found in both children with SCA and those with HbAA were distinct in this study. The I431V is an emerging mutation that was first described by Sutherland *et al*., in 2009, in isolates from British travellers, who visited Nigeria [14]. The I431V mutation has also been reported in other studies [6,15,22,23]. The similar prevalence of A581G, A613S and I431V mutations found in this study was also demonstrated in the study by Sutherland *et al*. 2009 [14]. The prevalence of the A437G mutation was the highest, with almost 100% prevalence in both study groups. A similar high prevalence was seen in Angola, Equitorial Guinea and Cameroon with a prevalence of up to 97.9%, 90.5% and 90% respectively [24-26]. This high prevalence may be due to the fact that the A437G mutation is the first point mutation to occur in *pfdhps* gene, and overtime this mutation has become fixed and stable in Africa.

Another important finding in this study was the complete absence of the *pfdhps* K540E mutation and the double mutant haplotype (A437G+K540E) in children with SCA and HbAA. This finding is in keeping with other studies done in West Africa, with low prevalence of K540E in children [27,28]. On the contrary, in Nigeria, Happi *et al*. in Ibadan, in 2004, reported a K540E prevalence of up to 24%. However, similar to our finding, Pearce *et al*. in 2009 in Nigeria, found a complete absence of the K540E mutation [29-30]. A K540E prevalence of 50% is the threshold above which the efficacy of SP as IPT in young children is compromised [11]. Therefore, the absence of the *pfdhps* K540E mutation in this study has significant implications that SP remains efficacious as IPT in infants and young children with SCA and those with HbAA. There were two major *pfdhps* mutant haplotypes found in this locale: the single mutant haplotype - ISGKAA and the quintuple mutant haplotype - VAGKGS. These mutant haplotypes were also the major mutant haplotypes described by Sutherland *et al*. 2009, Oguike *et al*., 2016 and Chao *et al*. 2020 in isolates from cases of imported malaria from Africa [6,14,15]. Similar to our study, the prevalence of the VAGKGS (80%) haplotype was higher than that of the ISGKAA haplotype (50%) found in the study by Sutherland *et al*. 2009 [14]. The fact that the prevalence of the *pfdhps* point mutations and mutant haplotype VAGKGS was high in this study, despite lack of direct exposure to SP in the study population (both those with SCA and HbAA), further confirms that the spread of antifolate resistance is not confined only to the population directly exposed to SP, but the entire population in a region where SP is used (such as in IPTp) is at risk of SP resistance [31].

The prevalence of *pfdhps* point mutations S436A, A581G and A613S were significantly higher in children with HbAA compared to those in children with SCA. This may mean that the homozygous sickle cell gene is associated with a lesser risk of selection or spread of these *pfdhps* point mutations compared to the normal homozygous Haemoglobin A gene. A multiple logistic regression model showed that the homozygous Haemoglobin A gene is a significant predictor of the presence of the *pfdhps* A613S mutation (Table 3). The prevalence of the quintuple mutant *pfdhps* haplotype (VAGKGS) was significantly higher in children with HbAA compared to that in children with SCA, while a significantly higher prevalence of the *pfdhps* single mutant haplotype (ISGKAA) was found in children with SCA compared to that in children with HbAA. In this study, multiple logistic regressions (Table 3) showed that HbAA was a significant predictor of the presence of the VAGKGS haplotype, which is 2.5 times as likely to occur in children with HbAA haplotype compared to those with SCA. Thus, it can be inferred that the normal haemoglobin AA allows for more selection of parasites with mutant *pfdhps* haplotype: VAGKGS (which indicates a high level of SP resistance), while the homozygous sickle cell gene protects against its selection.

More so, in this study, the GMPD in those with HbAA (3671.13±10.64) was significantly higher than (522.89±5.30) in those with SCA (t=-5.72, p < 0.001). According to the mechanism of spread of antimalarial resistance, resistant genes are transmitted in a population via gametocytes [31,32]. In children with SCA, spread of SP resistance may less likely occur, due to a lesser parasite biomass and gametocyte production, compared to those with HbAA. There was a significant relationship between mean parasite density and the type of *pfdhps* mutant haplotype in the study population; the highest GMPD was found in those with VAGKGS mutant haplotype, being significantly higher than the GMPD found in those with the single mutant haplotype: ISGKAA. This supports the mathemathical models of White *et al*. and Pongtavornpinyo *et al*. that the chance of selection and preferential survival of drug-resistant mutants from patients with high parasite density is greater than if a similar mutant arose in a person with much lower parasitaemia [16,17,31]. However, parasite density was not found to be a significant predictor for the occurrence of VAGKGS. This study has some limitations; it was done in a single locale (Benin City), thus it may be limited in describing the widespread distinct pattern and prevalence of the *pfdhps* mutations/mutant haplotypes in children with SCA and HbAA in Edo State and in Nigeria.

## Conclusion

The K540E mutation which is an indicator of significant resistance to SP leading to poor efficacy of SP-IPT in young children was totally absent in this study. Thus, there may be no significant resistance to SP in the study population, especially in those with SCA. Therefore, the use of SP as IPT can be explored in children younger than five years with SCA living in Benin City. The normal haemoglobin haplotype (HbAA), is a significant predictor of the occurrence of the quintuple mutant *pfdhps* haplotype: VAGKGS; while the homozygous haemoglobin sickle cell gene may confer protection against the occurrence and spread of mutations on the *pfdhps* genes in children with SCA.

### What is known about this topic

- Mutations on the Plasmodium falciparum dihydropteroate synthase (pfdhps) genes are the molecular markers of Plasmodium falciparum resistance to Sulphadoxine;
- Parasite densities/Biomass in persons with HbAA are higher than that in those with SCA;
- Mathematical models and theories show that resistant parasite strains are more likely to occur in infections with high parasite densities than in those with lower parasite densities.

### What this study adds

- This is the first study to demonstrate the mathematical model that selection of mutant haplotypes is more likely to occur in infections with high parasite density than in those with low parasite burden;
- The HbAA predisposes to the occurrence of the VAGKGS haplotype, while the HbSS phenotype may protect against the occurrence of pfdhps point mutations and the VAGKGS haplotype;
- The K540E mutation was totally absent in all samples in both study population (HbAA and SCA), thus the use of SP-IPT as malaria chemoprevention in children with SCA can be explored in Benin City.

## Annexes

Annex 1: types of mutant *pfdhps* haplotype found in study population and the corresponding combination of point mutations (PDF-335KB)

Annex 2: mean parasite density in study participants with different *pfdhps* haplotype (PDF-330KB)
